# Collaboration between grass seedlings and rhizobacteria to scavenge organic nitrogen in soils

**DOI:** 10.1093/aobpla/plu093

**Published:** 2015-01-06

**Authors:** James F. White, Qiang Chen, Mónica S. Torres, Robert Mattera, Ivelisse Irizarry, Mariusz Tadych, Marshall Bergen

**Affiliations:** Department of Plant Biology and Pathology, Rutgers University, New Brunswick, NJ, USA

**Keywords:** Grasses, microbiome, nitrogen use efficiency, oxidative nitrogen scavenging, plant growth-promoting rhizobacteria, symbiosis

## Abstract

Plants require nitrogen (N) to make proteins, nucleic acids and other biological molecules. It is widely accepted that plants absorb inorganic forms of N to fill their needs. However, recently it has become clear that plants also have the capacity to absorb organic N from soils. In this paper we describe a new kind of symbiosis involving seed-vectored rhizobacteria and grasses that is targeted at enhancing acquisition of organic N from soils. Our proposal is based on results of experiments on seedlings of grass species *Festuca arundinacea* Schreb., *Lolium perenne* L. and *Poa annua* L. that suggest: (i) seed-vectored rhizobacteria colonize seedling roots and influence their development; (ii) reactive oxygen secretion by seedling roots plays a role in organic N procurement by denaturing microbial proteins in the vicinity of roots (daytime activity); and (iii) plant root and microbial proteases degrade denatured proteins prior to absorption by roots (night-time activity). This research involved the following types of studies: (i) seedling root development experiments with and without rhizobacteria on a variety of substrates in agarose media and (ii) isotopic N-tracking experiments to evaluate the absorption into seedlings of N obtained from degradation of proteins. We hypothesize that grasses, in particular, are adapted to scavenge organic N from soils through application of this ‘oxidative nitrogen scavenging’ symbiosis with rhizobacteria, and their soil-permeating root systems. This newly discovered symbiosis in grass species could lead to new ways to cultivate and manage grasses to enhance efficiency of N utilization and reduce applications of inorganic fertilizers.

## Abstract (precis)

This article describes a process (termed ‘oxidative nitrogen scavenging’) where grasses scavenge organic nitrogen from microbes on and around roots. The authors propose a diurnal process where during the day roots produce and release hydrogen peroxide that oxidizes microbial exoenzymes around roots; at night hydrogen peroxide production ceases, then roots and symbiotic rhizobacteria secrete proteases that degrade the oxidized proteins to form peptides that are absorbed by roots. The existence of a mechanism for organic nitrogen scavenging in grasses emphasizes the nutritional importance of non-pathogenic microbes that associate with roots. Future applications of this process could result in new methods for the cultivation of crop plants.

## Introduction

All organisms require nitrogen (N) throughout development to make proteins, nucleic acids and other biological molecules. For plant growth, N is often the limiting nutrient. It has been believed that N available to plants was exclusively in the inorganic nitrate form. However, it has become clear that plants may obtain N from organic sources (Godlewski and Adamczyk 2007; Paungfoo-Lonhienne *et al*. 2008, 2012; Adamczyk *et al*. 2010). The need to push crop plants to higher yields has provoked heavy reliance on inorganic N fertilizers (Herridge *et al*. 2008). It has been estimated that only 30–50 % of applied inorganic N fertilizer is actually taken up by crop plants, with the rest lost in run-off, leaching or volatilization, increasing the probability for unintended environmental pollution (Mulvaney *et al*. 2009).

Agricultural scientists and plant breeders have targeted improvements in N use efficiency of plants as a way to reduce reliance on N applications (Garnett *et al*. 2009). Grasses associate with arbuscular mycorrhizae that function to absorb phosphorus and N from soils. Years of investigation on mycorrhizae have yielded volumes of information about the biology of mycorrhizal-plant associations (Gutjahr and Parniske 2013). However, comparatively little is known about other types of microbial endophytes or epiphytes that associate with healthy plants (Schulz and Boyle 2005). White *et al*. (2012) reported the occurrence of seed-vectored bacteria on many cool-season grasses. The bacteria, vectored on seed surfaces attached to adherent lemmas and paleas, rapidly colonize the roots of the seedlings after germination. The frequency of root colonizing bacteria on seeds of grasses suggested that bacteria could be involved in a nutritional symbiosis with plants; however, insufficient information was available to delineate clearly how the rhizobacteria benefited plants. In this paper we report studies conducted to evaluate the role of seed-vectored rhizobacteria in the nutrition of cool-season grass seedlings. In addition, we examine the role of grass root-secreted reactive oxygen in the process of protein degradation around roots. Some of the questions we pursued in this research include the following: Is there a survival/nutritional function for the seed-vectored bacteria on grass seeds? Do rhizobacteria facilitate organic N acquisition by grass seedlings? Does reactive oxygen secretion from grass roots relate to the function of seed-vectored bacteria?

## Methods

### Plant materials

Seeds used were selections of tall fescue (*Festuca arundinacea* Schreb.), perennial ryegrass (*Lolium perenne* L.) and annual bluegrass (*Poa annua* L.) obtained from the Rutgers University turf grass-breeding programme.

### Bacteria isolation and identification

To isolate bacteria, 40 seeds of each grass species were washed for 5 min with constant agitation in sterile water, then plated onto yeast extract-sucrose agar (YES) and 10 % tryptic soy agar (TSA; Difco). YES agar contained 1 % yeast extract (Difco), 1 % sucrose (Sigma-Aldrich, St Louis, MO, USA) and 1.2 % agar (Difco). Twenty seeds were plated on each medium (five seeds per plate) and incubated 1 week at laboratory ambient temperature. The most common colonies appearing were selected for sequencing of the 16S rDNA region. Bacterial DNA was extracted using the UltraClean^®^ Microbial DNA Isolation Kit (MoBio Laboratories, Carlsbad, CA, USA). The 16S rRNA gene was amplified using the universal primers 27F (5′-AGAGTTTGATCMTGGCTCAG) and 1492R (5′-CGGTTACCTTGTTACGACTT). Amplicons were sent to be sequenced by Genewiz (South Plainfield, NJ, USA). High-quality sequences were selected and bacteria were identified by searching for sequence similarity using BLAST (NCBI GenBank).

### Removal of bacteria from seeds

To conduct seedling development studies, seeds free of bacteria were prepared by surface disinfection. This was accomplished by agitating seeds on a rotary shaker in a 3 % sodium hypochlorite solution for 35–40 min, after which seeds were rinsed twice for 5 min in sterile water. We optimized the surface disinfection conditions so that seed germination frequency was not reduced but rhizobacteria no longer could be seen on or isolated from resulting seedlings.

### Preparation of media for seedling development experiments

Water agarose (0.7 %; Type 1 Low EEO, Sigma-Aldrich) was used as the base medium. This medium contained no added nutrient source and at 0.7 % agarose concentration was an ideal consistency for grass seedling root penetration. This base medium was amended using various vitamin or nutrient substrates (Tables 1–3). The following nutrient sources were employed in experiments: ammonium nitrate (Sigma-Aldrich), alanine (Sigma-Aldrich), biotin (Sigma-Aldrich), egg albumin (Sigma-Aldrich), catalase (Sigma-Aldrich), cellulase (Sigma-Aldrich), glycine (Sigma-Aldrich), lipase (Sigma-Aldrich), myo-inositol (Sigma-Aldrich), nicotinic acid (Sigma-Aldrich), pronase^®^ (Behring Diagnostics), sodium ascorbate (Sigma-Aldrich) and yeast extract (Sigma-Aldrich). Nutrient sources or vitamins were added to media after autoclaving, except where the protein is identified as denatured (Tables 2 and 3). Media was poured into 6 cm plastic Petri plates.

**Table 1.** Annual bluegrass (*Poa annua*) seedling root hair growth with and without seed-vectored rhizobacteria.

Treatment	Bacteria present		Bacteria absent	
	% Roots vertical	Root hairs	% Roots vertical	Root hairs
Agarose	100.00	+	11.12	−
Yeast extract (0.01 %)	91.67	+	75.00	+
Glycine (0.01 %)	88.89	+	0.00	−
Biotin (0.01 %)	81.25	+	8.69	−
Nicotinic acid (0.01 %)	61.54	+	0.00	−
Myo-inositol (0.01 %)	83.33	+	10.00	−
Lipase (0.1 %)	N/A (root inhibition)		64.10	+
Albumin (0.1 %)	N/A (root inhibition)		52.94	+
Pronase (0.1 %)	N/A (root inhibition)		82.05	+
Catalase (0.01 %)	N/A (root inhibition)		77.08	+
Lipase (0.1 %) + 0.01 % ascorbate (pH 5.6)	N/A		N/A	−
Lipase (0.1 %) + 0.001 % ascorbate (pH 5.4)	N/A		N/A	+

**Table 2.** Tall fescue (*F. arundinacea*) seedling root growth with and without seed-vectored rhizobacteria.

Treatment	Bacteria present		Bacteria absent	
	% Roots vertical	Root hairs	% Roots vertical	Root hairs
Agarose	87.50	+	16.67	−
Yeast extract (0.01 %)	94.12	+	87.50	+
Alanine (0.01 %)	87.50	+	6.67	−
Glycine (0.01 %)	93.75	+	87.50	+
Denatured albumin (0.1 %)	65.20	+	93.75	+
Ammonium nitrate (0.01 %)	93.75	+	0.00	−

**Table 3.** Perennial ryegrass (*L. perenne*) seedling root growth with and without seed-vectored rhizobacteria.

Treatment	Bacteria present		Bacteria absent	
	% Roots vertical	Root hairs	% Roots vertical	Root hairs
Agarose	77.78	+	18.75	−
Yeast extract (0.01 %)	75.00	+	70.58	+
Alanine (0.01 %)	70.84	+	11.12	−
Glycine (0.01 %)	72.23	+	26.67	−
Denatured albumin (0.1 %)	Root suppression		81.82	+
Ammonium nitrate (0.01 %)	87.50	+	21.05	−

### Seedling development experiments

Seeds with and without native bacterial populations were placed onto plates. For each treatment 8 plates per treatment were used with 10–15 seeds on each plate. Plates were incubated for 10 days at laboratory ambient temperature in a 12-h alternating light/dark cycle. Seedlings were examined and assessed for the percentage of seedlings with roots growing downward into agarose and whether downward growing roots showed development of root hairs (Tables 1–3). Any inhibition of root growth was also noted.

### Microscopic examination of roots

**Figure 1.**
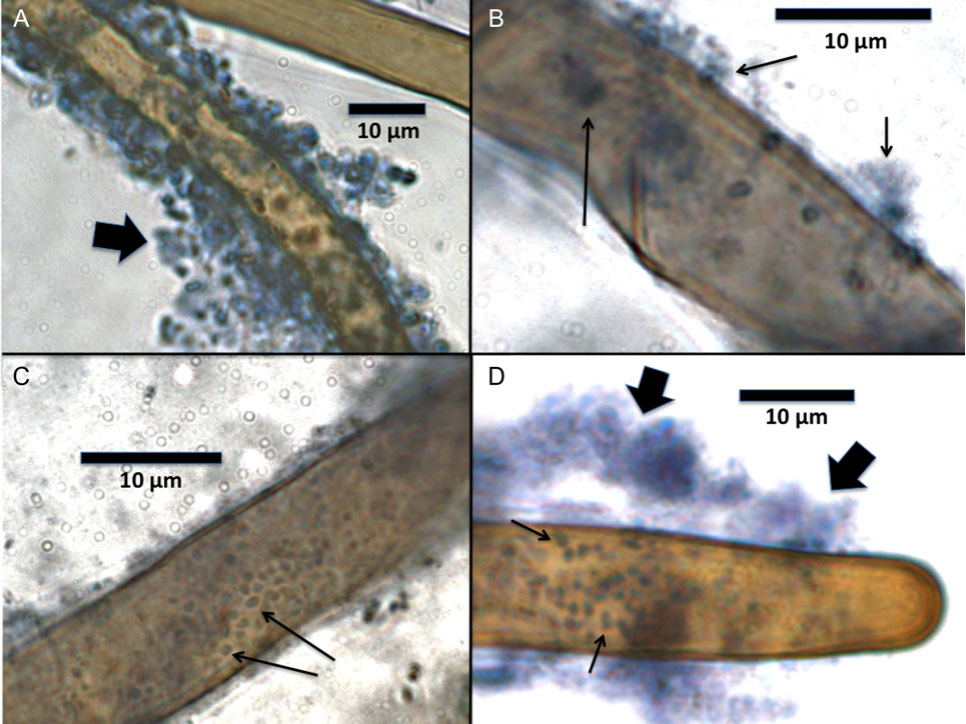
Bacteria on root hairs of cool-season grass seedlings; stained with DAB/peroxidase for reactive oxygen (brown) and counterstained with aniline blue/lacto-phenol for protein. (A) Bacteria (arrow) on surface of root hair of *L. perenne* seedling. (B) Bacteria and bacterial protein (arrows) on surface of root hair of *P. annua* seedling. (C) Bacteria (arrows) on surface of root hair of *P. annua* seedling. (D) Bacteria (small arrows) and denatured proteins (large arrows) on the surface of root hair of *P. annua* seedling.

**Figure 2.**
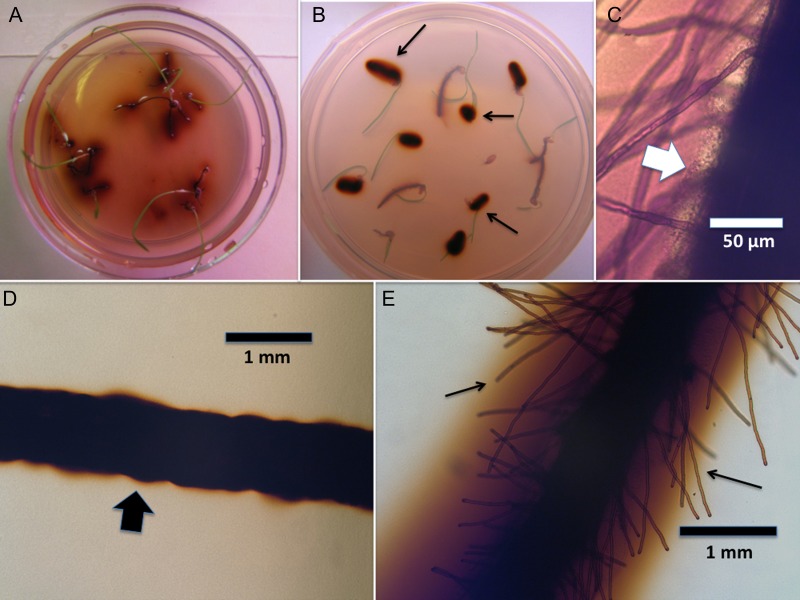
*Poa annua* seedlings showing reactive oxygen (H_2_O_2_) staining around roots. (A) Seedlings growing on 0.7 % agarose showing diffuse zones of reactive oxygen (brown) around roots. (B) Seedlings growing on 0.1 % albumin agarose showing dense zones of reactive oxygen (arrows) around roots. (C) Root surface showing root hairs and layer of bacteria (arrow). (D) Root without bacteria growing in 0.7 % water agarose medium, showing absence of root hairs. (E) Root with bacteria growing on 0.7 % water agarose medium, showing reactive oxygen zone and root hairs (arrows).

**Figure 3.**
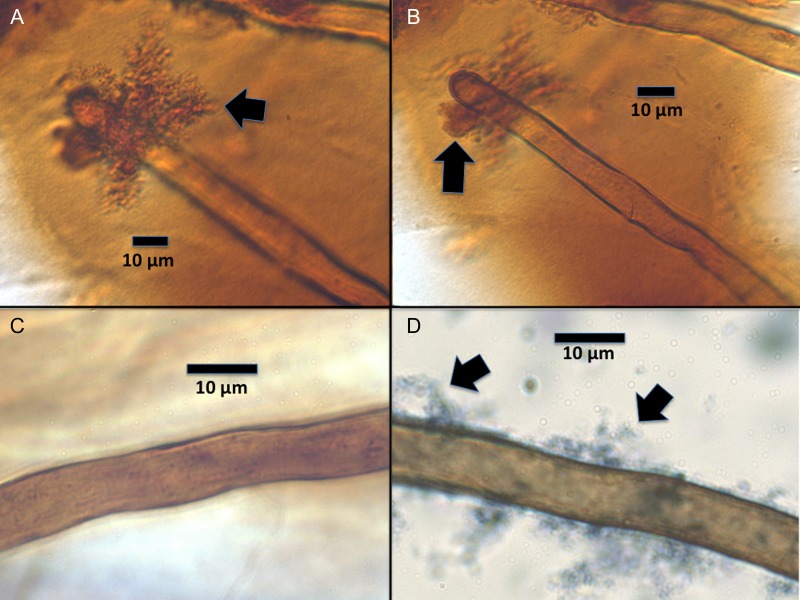
*Poa annua* root hairs stained for reactive oxygen. (A) Root hair showing denatured cellulase enzyme aggregation (arrow) on root hair with high reactive oxygen staining on protein as evidenced by darker brown colour. (B) Root hair showing the smoothing effect of the cellulase particle (arrow) in the area of close contact with the root hair. (C) Root hair without protein or bacteria. (D) Root hair showing adherent albumin protein (arrows).

For microscopic examinations (Figs 1–3), roots were stained for 8–10 h by flooding plates with 2.5 mM diaminobenzidine tetrachloride (DAB) and 5 purpurogallin units mL^−1^ of horseradish peroxidase (Type VI, Sigma-Aldrich) (White *et al*. 2012, 2014*b*). Stain was then poured off and plates rinsed several times with water. Seedling roots were examined microscopically using a compound light microscope through the reverse side of plates or by removing seedlings from agarose and placing in a drop of aniline blue/lacto-phenol stain on a slide (White *et al*. 2012, 2014*b*). For the evaluation of root effects on protein particles, examination was made only through the reverse of plates. DAB/horseradish peroxidase was used to stain H_2_O_2_ (red to brown colour); while aniline blue/lacto-phenol was used to stain proteins and bacterial cytoplasm (light blue to dense blue colour).

### Production of ^15^N-labelled protein mixture

To label bacterial proteins, *Bacillus amyloliquefaciens* (strain HF3) was inoculated into ^15^N-labelling medium composed of 1 % sucrose, Murashige and Skoogs salt base without nitrogen (Sigma-Aldrich), 0.02 % yeast extract and 0.16 % ^15^N-labelled glycine (Sigma-Aldrich) in 200 mL Erlenmeyer flasks. Flasks were then incubated at laboratory ambient temperature with constant agitation on a rotary shaker for 15 days. Cells were harvested from cultures by centrifugation and washed twice with phosphate-buffered saline solution. To extract proteins, cells were suspended in ice-cold acetone for 5 min, washed under nitrogen and then incubated in 1 % sodium dodecyl sulfate for 2 min. Extracted proteins were then freeze-dried to remove water; 0.16 g of labelled proteins was obtained. The labelled proteins were thoroughly mixed with 0.84 g of un-labelled egg albumin (Sigma-Aldrich) prior to use in media.

### ^15^N-labelled protein absorption experiment

**Figure 4.**
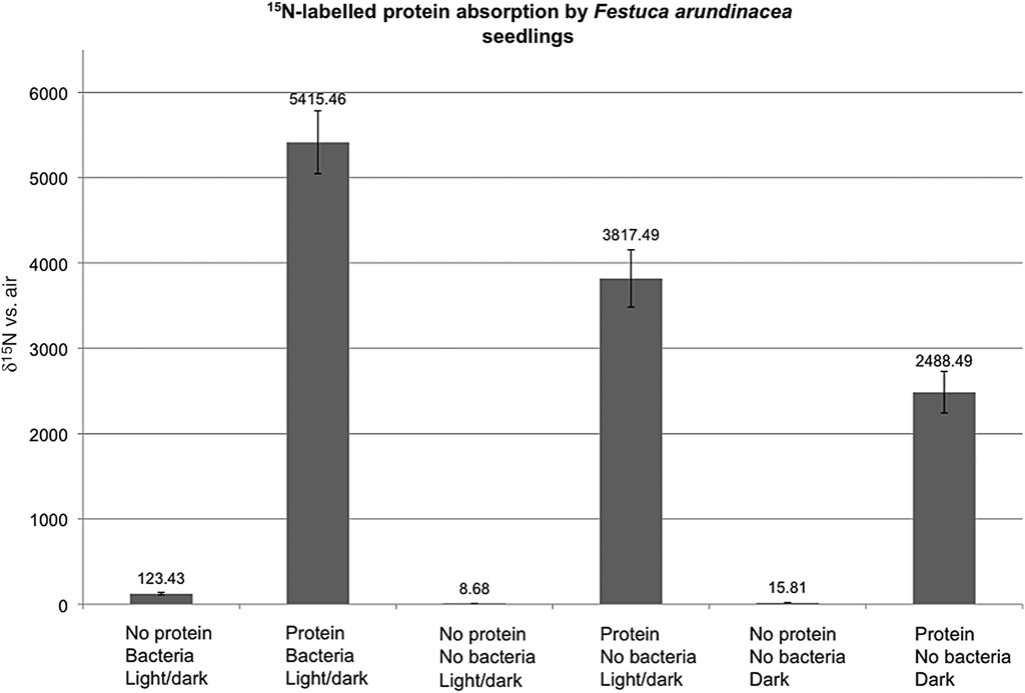
Results of ^15^N-labelled-protein absorption experiment showing enhanced ^15^N absorption due to rhizobacteria and reactive oxygen effects. All values expressed as mean ± standard error of mean; means with the same letter are not significantly different according to the Duncan's multiple range test (*P* < 0.05). The *y*-axis is the level of protein absorption (expressed as *δ*^15^N vs. air) and the *x*-axis shows the six treatments. The numbers above bars are means of the *δ*^15^N vs. air values and the bars show standard errors. The highest ^15^N incorporation (5415.46) was seen in shoots of seedlings bearing bacteria grown on agarose containing ^15^N-labelled protein in a 12-h alternating light/dark cycle (treatment = protein, bacteria, light/dark). Less ^15^N incorporation (3817.49) was seen in shoots of seedlings grown under the same conditions but without bacteria (treatment = protein, no bacteria, light/dark). Slightly less ^15^N incorporation (2488.49) was seen in shoots of seedlings grown on labelled proteins but without bacteria in total darkness to suppress reactive oxygen secretion (protein, no bacteria, dark). Minimal ^15^N incorporation was seen into shoots of seedlings grown on agarose that did not contain ^15^N-labelled protein (treatments = no protein, bacteria, light/dark; no protein, no bacteria, light/dark; no protein, no bacteria, dark).

Plastic Petri plates (6 cm diameter) containing either 0.7 % agarose or 0.7 % agarose with 0.05 % of the labelled-protein mixture were prepared. The labelled-protein mixture was added to agarose after autoclaving with the agarose at ∼70 °C and rapidly cooled to minimize additional protein denaturation. Twelve tall fescue seeds, either with seed-vectored rhizobacteria present or with rhizobacteria removed by surface disinfection as previously described, were placed onto plates of both media. The following treatments were prepared: (i) no protein in medium and seeds with rhizobacteria in 12-h alternating light/dark cycles; (ii) no protein in medium and no rhizobacteria on seeds in 12-h alternating light/dark cycles; (iii) protein in medium and rhizobacteria on seeds in 12-h alternating light/dark cycles; (iv) protein in medium but seeds without rhizobacteria on seeds in 12-h alternating light/dark cycles; (v) no protein in medium and no rhizobacteria on seeds maintained in total darkness; and (vi) proteins in medium but seeds without rhizobacteria and maintained in total darkness. Three to six replicate plates were made for each treatment (Fig. 4). Plates in dark treatments were wrapped in aluminium foil to exclude light. All plates were placed in a clear plastic sealable canister and placed under fluorescent lights at laboratory ambient temperature for a 10-day period. After the incubation period, the shoots were excised from seedlings, with all shoots on a plate being combined and dried in an oven at 60 °C overnight. After drying, samples were further processed and analysed for ^15^N/^14^N ratio using mass spectroscopy at the Stable Isotope Laboratory at the Odum School of Ecology at the University of Georgia, Athens, GA, USA. Results were expressed as *δ*^15^N vs. air measurements (Fig. 4). Statistical analysis of data involved application of the Duncan's multiple range test (*P* < 0.05).

## Results

### Identification of culturable bacteria

Culturable seed-vectored bacteria from tall fescue include *Pseudomonas* sp. (JX089400; 100 % identical) and *Pantoea agglomerans* (JX089401; 100 % identical); from perennial ryegrass *B. amyloliquefaciens* (KP053610; 99 % identical) and *Staphylococcus epidermidis* (KP053611; 99 % identical); and from annual bluegrass *Paenibacillus hordei* (KJ528493; 98 % identical) and *Pantoea* sp. (KJ528494; 99 % identical).

### Seedling development experiments

Seedlings that possessed the rhizobacteria showed a majority of the seedling roots to be downward oriented with abundant root hair growth (Tables 1–3). Removal of rhizobacteria from surfaces of seed coats resulted in anomalous root development on agarose without nutrients. Without bacteria, the majority of the seedling roots grew on the surface of the agarose, and any roots that grew downward into the agarose lacked root hair development. Substances that were found to consistently restore normal root development in bacterial-free seedlings included yeast extract and various proteins (Tables 1–3). In tall fescue the amino acid glycine was seen to restore seedling development, but this was not seen in the other grasses (Tables 1–3). When rhizobacteria were present on seedlings, protein levels that stimulated proper seedling root development in seedlings without rhizobacteria were instead inhibitory (Table 2). The addition of the reactive oxygen scavenger sodium ascorbate to agarose-containing proteins was seen to suppress the capacity of the proteins to trigger proper seedling root development in the absence of rhizobacteria (Table 1). The addition of ammonium nitrate to agarose was not sufficient to restore proper seedling root development in the absence of rhizobacteria (Tables 2 and 3).

### Visualization of rhizobacteria and proteins on roots

Rhizobacteria could be visualized adhering closely to the surfaces of root hairs (Fig. 1A–D). Proteins could also be visualized in the vicinity of bacteria (Fig. 1B and D). DAB/horseradish peroxidase staining showed that H_2_O_2_ was abundantly secreted into agarose from roots (Fig. 2A). In media that contained protein H_2_O_2_ staining appeared to be more intense than in agarose without protein (Fig. 2B). H_2_O_2_ appeared to be secreted from the central axis of the seedling roots, rather than from root hairs (Fig. 2C–E). Visual evidence of root protease activity was seen in the smoothing of protein crystals that were in contact with root hairs (Fig. 3A and B). Seedlings grown on protein media frequently had protein accumulations on root hair surfaces (Fig. 3C and D).

### ^15^N-labelled-protein absorption experiment

The protein mix itself had a *δ*^15^N vs. air of 8869.57, indicating that it contained a substantial amount of ^15^N-labelled proteins. The untreated tall fescue seed was 0.71 ± 0.04 *δ*^15^N vs. air (all *δ*^15^N vs. air values expressed as mean ± standard error of mean). In the experiment, the lowest absorption of ^15^N from labelled protein (2488.49 ± 243.85 *δ*^15^N vs. air) was seen in seedling shoots of tall fescue without rhizobacteria cultured in constant darkness in order to eliminate H_2_O_2_ secretion from roots (Fig. 4). The highest level of ^15^N absorption from ^15^N-labelled proteins (5415.46 ± 367.93 *δ*^15^N vs. air) was seen in seedling shoots with rhizobacteria cultured under alternating light/dark conditions (Fig. 4). Seedling shoots without rhizobacteria grown in alternating light/dark conditions were intermediate (3817.49 ± 336.4 *δ*^15^N vs. air) between the previous two treatments (Fig. 4). Minimal incorporation of ^15^N into shoots was seen in the treatments without rhizobacteria grown on agarose without proteins (Fig. 4). Analysis of shoots of seedlings with rhizobacteria on water agarose free of any proteins showed several times more incorporation of the ^15^N label (123.43 ± 14.4 *δ*^15^N vs. air) compared with seedlings without bacteria under the same conditions (8.68 ± 1.31 *δ*^15^N vs. air) (Fig. 4).

## Discussion

### Organic N absorption by roots

Previous research has shown that grasses, and plants in general, secrete proteases from their roots. Adamczyk *et al*. (2008, 2010) conducted studies on plant growth on proteins and presented evidence that plants actively degrade proteins without microbes. Further, Paungfoo-Lonhienne *et al*. (2008) showed that certain plants were capable of degrading proteins on the surfaces of root cells. Results in our seedling protein absorption experiment may reflect the activities of both plant and rhizobacterial proteases. The lowest ^15^N absorption into seedling shoots on media with proteins was in the seedlings without rhizobacteria maintained in darkness (Fig. 4). In darkness, H_2_O_2_ secretion is minimized and absorption of organic N may reflect the activity of plant root proteases alone. A 53.4 % increase in incorporation of ^15^N into shoots was observed in seedlings maintained under alternating light/dark conditions without microbes where H_2_O_2_ was secreted from roots in the light periods. The importance of reactive oxygen secretion from roots to the process of N scavenging may be seen in this 53.4 % increase in N absorption from protein over that seen in the seedlings without bacteria maintained in darkness where reactive oxygen was not secreted from roots. In our experiment, the real benefit of reactive oxygen may be lower than that seen in nature because our proteins were already partially denatured by the use of sodium dodecyl sulfate during the extraction process. The role of reactive oxygen in the process of protein degradation was further emphasized by results of the seedling development experiments (Table 1), where the addition of the antioxidant sodium ascorbate to agarose containing the enzyme lipase was seen to suppress development of root hairs. With reduced oxidative denaturing of lipase, the plant proteases alone were insufficient to degrade enough of the enzyme to trigger root hair development. Additional experiments will be needed to get a more accurate assessment of the real value of the pre-oxidation of proteins in the degradation process. The importance of the rhizobacteria to the process of N scavenging from proteins is evident in the 41.86 % increase in incorporation of ^15^N into shoots of microbe bearing seedlings incubated in alternating light/dark conditions over that of seedlings without microbes under the same conditions. Secretion of H_2_O_2_ from roots and the added degradative capacity of the rhizobacteria increased organic N acquisition by seedlings ∼119.74 % over what was seen in the seedlings without microbes or secreted H_2_O_2_. Evidence for the direct action of proteases produced by grass roots may be seen not only in accumulation of ^15^N in shoots in the bacterial-free seedlings grown on the ^15^N-labelled proteins, but also in the smoothing of protein crystals in direct contact with grass seedling root hairs (Fig. 3A and B). There is evidence that some plants (e.g. rice and wheat) under drought stress enhance their efforts to scavenge organic N from soils, where it has been shown that secreted proteases are up-regulated in plants under water stress (Kohli *et al*. 2012). The trigger for the shift to organic N use may be restriction of access to mineralized N due to reduced water flow through soils (Kohli *et al*. 2012).

### Seedling development and rhizobacteria

The importance of the role of the rhizobacteria to nutrient acquisition by grass seedlings is emphasized in the results of seedling root development experiments with and without rhizobacteria on various substrates (Tables 1–3). Growth of seedlings with and without bacteria on nutrient-free agarose showed that bacteria stimulated roots to form root hairs and trigger the root gravitropic response where roots grew downward into the medium (Tables 1–3). In the absence of rhizobacteria, roots did not develop properly; roots tended to remain on the agarose surface and any roots that did penetrate into media did not produce root hairs. Seedling roots of all three species of grasses responded similarly when germinated on water agarose medium. It seems evident that grass seedlings interact with rhizobacteria in some way that triggers proper seedling root development. In seedlings without rhizobacteria, yeast extract and various proteins could consistently restore proper root development in triggering root hair formation and downward root growth. While we have no data that can clarify the molecular communication between rhizobacteria and grass seedling, the capacity of organic N to restore proper root development suggests that the function of rhizobacteria relates to organic N acquisition. This role is further emphasized because protein levels that were sufficient to stimulate root hair development without the presence of rhizobacteria were inhibitory to seedling growth when bacteria were present (Tables 1 and 3). Rhizobacteria appear to significantly enhance N scavenging capacity of grasses as is evident in results of the ^15^N-labelled-protein absorption experiment (Fig. 4). Changes in plant development have also been shown for rice plants containing certain fungal endophytes (Rodriguez *et al*. 2009). It is entirely possible that improved growth in plants bearing other types of non-pathogenic microbes could also relate to increased supply of organic nutrients, with microbes functioning as collaborators of plants in scavenging organic N from soils or rhizosphere microbes.

### Adaptations in grasses

The developmental dependence of grass seedling roots on the rhizobacteria suggests that grasses have evolved to rely on these seed-vectored rhizobacteria in order to maximize scavenging of organic N. Features of development of grasses seem consistent with this idea. Grasses possess lemmas and paleas that adhere tightly to the surface of the seed coat. We suggest that the adherent plant tissues are adaptations for vectoring the rhizobacteria on the seed surface. It is apparent that rhizobacteria are vectored on the seed surface because rigorous surface disinfection of seeds is sufficient to eliminate rhizobacteria from seedlings. Microbes vectored in and on seeds are likely to be essential elements of a plant's microbiome that must be transmitted reliably from parent to offspring. The bacteria that are vectored on seeds of the grasses that we examined are likely essential, since seedlings do not develop properly without them. It seems very likely that the rhizobacteria are important components of the ‘functional microbiome’ of grasses (Lakshmann *et al*. 2014; White *et al*. 2014*a*). Without competent rhizobacteria seedlings would likely be at a competitive disadvantage in terms of nitrogen supply.

### Oxidative nitrogen scavenging

In a previous study we observed degradation of some of the bacteria on surfaces of grass seedling roots (White *et al*. 2012). We hypothesized that the direct oxidative degradation of bacteria and their proteins on roots could provide a source of N for plants, and we termed the process oxidative N scavenging (ONS). Our results here suggest that ONS involves the rhizobacteria integrally, and rather than being the exclusive target of ONS, they are crucial in its operation in providing additional proteases that efficiently degrade oxidatively denatured proteins. It seems likely that all microbial exoenzymes in the vicinity of roots would be vulnerable to degradation through ONS by grasses.

### Diurnal model of ONS in grasses

**Figure 5.**
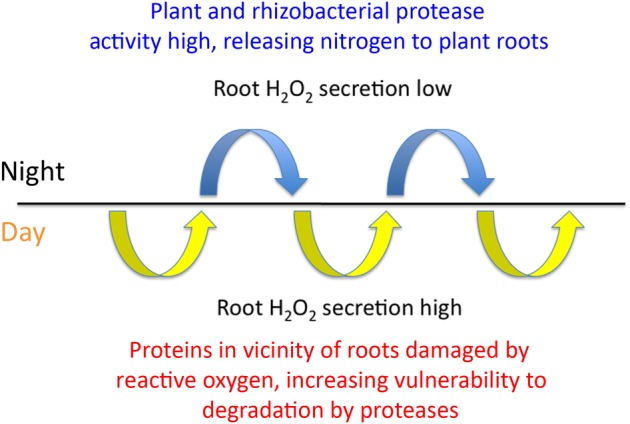
Proposed cyclic model of the oxidative nitrogen scavenging process in grasses. In daylight grasses secrete hydrogen peroxide from roots in order to denature proteins (microbial exoenzymes) around roots. At night plant and microbial proteases degrade oxidized proteins to form smaller peptides or oligopeptides that may be absorbed by roots.

We suggest a diurnal nature to ONS in grasses, with oxidative protein denaturation occurring only during the day and denatured protein degradation and absorption occurring at night (Fig. 5; White *et al*. 2012). This results in a temporal separation of oxidative and degradative processes. This temporal separation of oxidative and degradative processes is necessary because proteases of the plant and rhizobacteria are also vulnerable to oxidation by H_2_O_2_. In this cyclic process, grass seedlings collaborate with seed-vectored rhizobacteria to scavenge organic N in the vicinity of roots. Both grass and rhizobacteria have active roles in the decomposition of proteins around roots. Grass roots secrete reactive oxygen that denatures proteins, while rhizobacteria and grasses secrete proteases that further degrade proteins into smaller peptides or oligopeptides that may be absorbed by grass roots. Denaturation of proteins by reactive oxygen involves protein unfolding and increased exposure of peptide bonds to proteases (Fligiel *et al*. 1984; Davies 1987). Denatured proteins become highly susceptible to degradation by proteases (Davies 1987). Bongarzone *et al*. (1995) found enhanced susceptibility of peroxidized myelin proteins to degradation by *Bacillus* protease subtilisin. Several of the seed-vectored bacteria we isolated from grasses are known to produce secreted proteases, including *B. amyloliquefaciens*, *Paenibacillus hordei*, *Pantoea agglomerans* and *S. epidermidis* (Wells *et al*. 1983; Bott *et al*. 1988; Rodarte *et al*. 2011; Kim *et al*. 2013; Sugimoto *et al*. 2013). It is interesting to speculate that grasses may manage the rhizosphere to maximize protein production by rhizosphere microbes. The secretion of complex root exudates composed of carbohydrates and proteins into the rhizosphere could be targeted at encouraging secretion of microbial enzymes to degrade the exudate components. In essence, grasses may be cultivating microbes in the rhizosphere and harvesting their proteins to support growth (Walker *et al*. 2003). Validation of the diurnal oxidative nitrogen scavenging process could provide a key to understanding the mystery of why many plants grow faster during the night (Nusinow *et al*. 2012). The absorption of protein fragments during the evening hours could provide the rapidly acquired nitrogenous nutrients plants require to build enzymes needed for the growth spurt that occurs in the predawn hours.

### Evidence for enhanced absorption of N from air

The results of the ^15^N-labelled protein absorption experiment (Fig. 4) suggests that the seed-vectored rhizobacteria not only enhance absorption of organic N through increased degradation of proteins, but they may also increase N available to plants by scavenging it from air. In this experiment, Petri plates containing agarose media and seedlings were maintained together in a closed plastic canister. We hypothesize that the degradation of proteins by plants and microbes released ^15^N-labelled ammonia or other compound into the air of the canister. Analysis of shoots of seedlings with rhizobacteria on water agarose free of any proteins showed a 14-fold increase in N absorption over that of seedlings grown under the same conditions but without rhizobacteria (Fig. 4). This difference can only be explained if rhizobacteria facilitated absorption of a ^15^N-labelled volatile in the canister. It should be noted that seedlings without bacteria absorbed some ^15^N as well. This is evident because seedlings without rhizobacteria or protein showed *δ*^15^N vs. air values of 8.68 ± 1.31 (light) and 15.81 ± 2.23 (dark), while seed samples used in this experiment showed a 0.71 ± 0.04 *δ*^15^N vs. air value and the agarose media showed no detectable N on analysis. It seems likely that the rhizobacteria are more efficient at capturing N than seedlings alone. Hurek *et al*. (1988) described a similar phenomenon in a study of bacteria associated with roots of kallar grass, where it was found that ammonia and nitrous oxide were being scavenged from the air by root-associated bacteria. Future experiments are needed to further evaluate the capacity of grass rhizobacteria to absorb and transfer to plants gaseous forms of N.

## Conclusions

This paper presents observations and experiments that support occurrence of an organic N scavenging process that we hypothesize functions daily in grasses. It is necessary to rigorously evaluate whether ONS, as we have outlined it based on laboratory studies, is actually occurring in grasses in soils. This confirmation would likely involve larger scale N tracking experiments. It is necessary to elucidate the culturable and non-culturable rhizobacteria that assist plants to scavenge N. Each bacterium will need to be evaluated for its role in the process of N scavenging. It is possible that the bacteria that were most active in N scavenging were not cultured, or perhaps are not culturable. In most cases we can isolate multiple species of bacteria from grass seeds and seedling roots. It is unknown whether the rhizobacteria function in consortia, or if only a single species is functional. Finally, there are some important applications that can come from this research. If there are rhizobacteria that are more efficient at degrading soil proteins, it may be possible to use them to enhance the N scavenging efficiency of commercial grasses. This could have widespread positive effects, both economically and environmentally in reducing costs to apply fertilizers and reduce inorganic N applications that contaminate bodies of water. Further, Beltran *et al*. (2014) demonstrated that *Agave tequilana* plants are capable of absorption of N from ^15^N-labelled bacteria, indicating that it is also capable of scavenging N from microbes. White *et al*. (2014*b*) demonstrated that reactive oxygen was associated with microbes in roots of seedlings of several species in other plant families, suggesting that the ONS process could be widespread in plants. A more complete understanding of which plants engage in N scavenging and exactly how it works in those plants could result in new approaches to cultivation and nourishment of crop plants.

## Sources of Funding

This research was supported by funds from the John E. and Christina C. Craighead Foundation, the Rutgers Turfgrass Science Center, USDA-NIFA Multistate Project W3147 and the New Jersey Agricultural Experiment Station.

## Contributions by the Authors

J.F.W. conceived the study; all authors contributed to experimental design; Q.C., I.I. and M.S.T. sequenced isolates, and M.T., R.M. and M.B. helped with experiments.

## Conflicts of Interest Statement

None declared.
